# Frequency of diarrhea in novel coronavirus 2019 infection 

**Published:** 2020

**Authors:** Rujittika Mungmunpuntipantip, Viroj Wiwanitkit

**Affiliations:** 1 *26 Medical Center, Bangkok Thailand*; 2 *Honorary professor, Dr DY Patil University, Pune, India; visiting professor, Hainan Medical University, Haikou, China *

**To The Editor**

Novel coronavirus 2019 infection is a new coronavirus disease (1). This disease started in China then it is imported to several countries. As a new disease, knowledge on its clinical feature is limited. In additional to respiratory manifestation, atypical clinical presentation of the new disease is possible. An important atypical clinical presentation is diarrhea. Here, the authors performed a summative analysis on available data on 314 cases of novel coronavirus 2019 infections (2 – 4) to calculate for frequency of diarrhea. Of those 140 cases, diarrhea is presented in 3 cases. The frequency of diarrhea is 2.14 % (95 % confidence interval = 0.70 % - 6.56 %). Based on this observation, it can show that diarrhea is a possible atypical clinical presentation of novel coronavirus 2019 infection. in fact, diarrhea is usually a possible but forgotten clinical presentation in new emerging disease such as swine flu (5). Practitioner should recognize the possibility that diarrhea might be the first clinical presentation of the patient with novel coronavirus 2019 infection. Nevertheless, the exact pathophysiology of diarrhea in novel coronavirus 2019 infection is unknown. Further study on this issue is recommende. 
